# 913. Lower Income Level Is Associated with Worse Physical and Social Functional Outcomes Among Burn Survivors

**DOI:** 10.1093/jbcr/irag033.438

**Published:** 2026-04-06

**Authors:** Sarah Wang, Amila Adili, Trevor A Pickering, Carly Marincasiu, Kimberly Roaten, Haig A Yenikomshian

**Affiliations:** Keck School of Medicine, University of Southern California, Los Angeles, CA; Southern California Clinical and Translational Science Institute (SC-CTSI), Keck School of Medicine, University of Southern California, Los Angeles, CA; Department of Population and Public Health Sciences, Keck School of Medicine, University of Southern California, Los Angeles, CA; Department of Surgery, University of Washington Medicine Regional Burn Center, Seattle, WA; Department of Psychiatry, University of Texas Southwestern Medical Center, Dallas, TX; Department of Psychiatry, University of Texas Southwestern Medical Center, Dallas, TX

## Abstract

**Introduction:**

Socioeconomic status, particularly income level, is increasingly recognized as a key determinant of long-term recovery following burn injury. Prior studies have established that lower income is associated with decreased likelihood of returning to pre-injury productivity levels. However, less literature has focused on its effect on patient reported outcomes. The aim of this study is to investigate the impact of income on various physical and social functional outcomes, to inform solutions that address systemic gaps in access to care.

**Methods:**

Data was collected from burn participants within a multi-center longitudinal database from 2015 to 2024. Primary outcomes were collected at 12 months after injury and included total family income, physical and social functional survey variables, and employment. Participants were divided into three groups based on pre-injury family income: lower income (<$50 K), moderate income ($50 K–$150 K), and higher income (>$150 K). The association between income level and outcomes was modeled via multivariable regression controlling for patient demographics (age, sex, race, education) and injury characteristics (%TBSA, inhalation injury).

**Results:**

Of the 1116 burn patients included, 637 (57.1%) were categorized as lower income, 373 (33.4%) were moderate income, and 106 (9.5%) were higher income. After controlling for confounders, the lower income group had significantly worse self-reported outcomes at follow-up compared to the higher income group in the following scales: PROMIS Ability to Participate in Social Roles (β = -4.25, *p*=.013), PROMIS Physical Function (β = -4.10, *p*=.006), and Satisfaction with Life (β = -3.56, *p*=.014). Furthermore, the lower income group had significantly lower odds of being employed at follow-up (OR = 0.19, *p*<.001). There were no significant differences between the moderate and higher income groups (Table 1).

**Conclusions:**

This study confirms that pre-injury lower family income is an independent predictor of worse physical and social outcomes following burn injury. Long-term recovery is significantly compromised by lower income, and targeted strategies are needed to address the systemic barriers that disproportionately affect this vulnerable population. Future research should investigate potential mediating factors, such as reduced access to rehabilitation resources or barriers to attending follow-up appointments, to clarify the relationship between socioeconomic status and compromised recovery.

**Applicability of Research to Practice:**

The implementation of targeted support services, such as financial counseling, vocational rehabilitation, social work, and mental health resources, may help mitigate disparities in burn recovery outcomes.

**Funding for the study:**

The contents of this abstract were developed under a grant from the National Institute on Disability, Independent Living, and Rehabilitation Research (NIDILRR grant number 90DPBU0007) and from the National Center for Advancing Translational Science (NCATS grant number UL1TR001855). NIDILRR is a Center within the Administration for Community Living (ACL), Department of health and Human Services (HHS). The contents of this abstract do not necessarily represent the policy of NIDILRR, ACL, HHS, or NIH and you should not assume endorsement by the Federal Government.

